# PD-L1 Is Expressed and Promotes the Expansion of Regulatory T Cells in Acute Myeloid Leukemia

**DOI:** 10.3389/fimmu.2020.01710

**Published:** 2020-07-31

**Authors:** Yuqing Dong, Yixiang Han, Yisha Huang, Songfu Jiang, Ziyang Huang, Rongrong Chen, Zhijie Yu, Kang Yu, Shenghui Zhang

**Affiliations:** ^1^Wenzhou Key Laboratory of Hematology, Department of Hematology, The First Affiliated Hospital of Wenzhou Medical University, Wenzhou, China; ^2^Central Laboratory, The First Affiliated Hospital of Wenzhou Medical University, Wenzhou, China; ^3^Division of Clinical Research, The First Affiliated Hospital of Wenzhou Medical University, Wenzhou, China

**Keywords:** acute myeloid leukemia, regulatory T cells, PD-L1, PD-1, interleukin-35

## Abstract

Intratumoral accumulation of CD4^+^CD25^+^Foxp3^+^ regulatory T (Treg) cells occurs in acute myeloid leukemia (AML), but little is known about the role of tumor cells themselves in this process. Here, we showed that an immune checkpoint PD-L1 expressed by AML cells promoted the conversion and expansion of Treg cells sustaining high expression of Foxp3 and PD-1 as well as a suppressive function. Furthermore, an AML cell line HEL overexpressed PD-L1 promoted the conversion and expansion of Treg cells and CD4^+^PD-1^+^Foxp3^+^ T (PD-1^+^Treg) cells from the conventional CD4^+^ T cells. CD4^+^CD25^high^PD-1^+^ T cells secreted more IL-10 production than CD4^+^CD25^high^PD-1^−^ T cells. IL-35, another cytokine secreted by Treg cells, promoted the proliferation of HL-60 cells and enhanced chemoresistance to cytarabine. Blockade of PD-1 signaling using anti-PD-L1 antibody dramatically impaired the generation of Treg cells and sharply retarded the progression of a murine AML model injected with C1498 cells. The frequency of intratumoral PD-1^+^ Treg cells was capable of predicting patient survival in patients with AML. In conclusion, our data suggest that PD-L1 expression by AML cells may directly drive Treg cell expansion as a mechanism of immune evasion and the frequency of PD-1^+^ Treg cells is a potential prognostic predictor in patients with AML.

## Introduction

The standard treatments for acute myeloid leukemia (AML) have remained practically unchanged over the past 40 years (1, 2), demonstrating that despite advances in our understanding of the etiology of AML, a more thorough knowledge of the biology of AML is still required in order to develop better prognostication tools and rationally design more efficient therapies. The clinical signs and symptoms of this disease and the ultimate therapeutic effect rely mainly on the biological characteristic of tumor cells themselves (3). Another key factor in the pathogenesis and treatment of this disease is the immune system. Despite a significant number of studies have demonstrated a highly immunosuppressive tumor microenvironment in the bone marrow (BM) of patients with AML (4), the mechanisms by which AML blasts create an immune-privileged niche and restrain immune response are poorly understood.

Regulatory T (Treg) cells and myeloid-derived suppressor cells are two leading components of the immune suppressive tumor microenvironment. Elevated Treg cells are presented in the circulating and BM microenvironment in both AML patients and AML-bearing mice model (5, 6). Furthermore, these Treg cells infiltrated in the tumor microenvironment exhibit stronger suppressive abilities than normal circulating Treg cells (5). Tregs cells consist mainly of natural and induced Treg cells, and these two subsets may have different biological characteristics. It has been recognized that more induced Treg cells exist in the tumor microenvironment (7, 8). Although many molecules including indoleamine 2,3-dioxygenase (IDO) (9) in the BM microenvironment have been reported to induce the generation of Treg cells, this phenomenon has not be fully explained.

The interaction of AML cells and Treg cells may induce the generation of Treg cells. PD-L1^−/−^ antigen-presenting cells rarely convert naïve CD4 T cells to induced Treg cells (10), suggesting the necessary role of PD-L1 for the induction of Treg cells. Overexpression of PD-L1 in human solid cancers is frequently observed and associated with unfavorable clinical outcomes (11). PD-L1 binds to its receptor PD-1 on activated T cells to suppress anti-tumor immunity by counteracting T cell-activating signals (12). Although the expression patterns of the PD-L1 protein in AML cells are considerably controversial (13, 14), there is no doubt that PD-L1 can be induced by some cytokines in AML cells and in the BM microenvironment of AML patients (15). In these cytokines, IFN-γ produced by effector T cells is the major cytokine to induce the expression of PD-L1 (16). Other molecules within tumor microenvironment such as CXCL5 (17), HMGB1 (18), and VEGF (19) also up-regulate the expression of PD-L1. It has been reported that PD-1 expressed in Treg cells plays a critical role in regulating peripheral immune response (20, 21). Treg cells with upregulated PD-1 had a stronger suppressor function during chronic infection (21). Whether Treg cells with PD-1 expression in the tumor microenvironment also exhibit a stronger inhibitory function remains uncharacterized.

In the present study, we found that CD4^+^CD25^+^FoxP3^+^ (Treg) and CD4^+^FoxP3^+^PD-1^+^ (PD-1^+^Treg) cells were enriched in BM microenvironment of patients with AML and exhibited a stronger inhibitory ability against effector T cells. Anti-PD-L1 antibody inhibited the generation of PD-1^+^ Treg cells and retarded AML development in a murine model. The frequency of BM-infiltrating PD-1^+^Treg cells might be a potential prognostic predictor in AML patients.

## Materials and Methods

### Enrolled Patients

A total of 65 patients with newly diagnosed AML excluding APL with *t*(15, 17)_(*q*22;*q*12)_; PML-RARA, including 36 males and 29 females with a median age of 48 years old (range: 17–76) were enrolled at Department of Hematology of the First Affiliated Hospital of Wenzhou Medical University (Table S1). The diagnosis and classification of AML were established and performed according to the 2016 WHO Classification for AML (22). All patients must have completed at least a cycle of induction chemotherapy, and the median follow-up time was 15 months (range: 1–55). The control group consisted of 10 age- and gender-matched healthy donors, which included 6 males and 4 females from 17 years old to 58 years old, with a median age of 41 years. This study received approval from the Institutional Ethics Committee of the First Affiliated Hospital of Wenzhou Medical University, and all participants signed written informed consent in accordance with the Declaration of Helsinki. Ficoll-hypaque density gradient centrifugation were used to isolate peripheral blood mononuclear cells (PBMNCs) and BM mononuclear cells (BMMNCs).

### Isolation and Culture of Treg Cells and Effector T Cells

PD-1^+^CD4^+^CD25^high^ T cells, PD-1^−^CD4^+^CD25^high^ T cells, and CD4^+^CD25^−^ effector T cells were isolated from BMMNCs of 4 patients with AML by FACSAria III (Becton Dickinson, San Jose, CA, USA). The suppressive function of PD-1^+^CD4^+^ CD25^high^ T cells, PD-1^−^CD4^+^CD25^high^ T cells were determined using mixed leukocyte culture assay according to our previously reported procedure (6).

CD4^+^ T cells were selected from PBMNCs of healthy donors using MACS CD4^+^ T cell isolation kit (Miltenyi Biotec, Bergisch Gladbach, Germany) and subsequently seeded at 5 × 10^4^ cells/well on 96-well plates coated with anti-CD3 monoclonal antibody (1 μg/ml) and stimulated with anti-CD28 monoclonal antibody (3 μg/ml) (both from eBiosciences, San Diego, CA, USA) and 20 ng/ml IL-2 for 5 days.

Full-length hPD-L1 cDNA was cloned into the lentivirus expression vector CMV-MCS-PGK-Puro, which was tranfected simultaneously with three pakaging vectors into 293FT cells to obtain virus particles. HEL cells were infected with these virus particles using pLX, and were subsequently screened with 800 μg/ml G418 for 5 days.

### Quantitative RT-PCR for Gene Expression Analysis

PD-L1 mRNA expression of BMMNCs from between healthy donors and patients with AML was performed according to our previously reported method (6). PCR analysis of PD-L1 was performed using the primer pairs as follow: PD-L1-forward: 5′- GTGCCGACTACAAGCGAATT-3′ and -reverse: 5′- CTTGGAATTGGTGGTGGTGG-3′; GAPDH-forward: 5′-ATCATCAGCAATGCCTCC-3′ and -reverse: 5′-CATCACGCCACAGTTTCC-3′.

### Cytokine Analysis

The PD-1^+^CD4^+^CD25^high^ T cells and PD-1^−^CD4^+^CD25^high^ T cells were cultured in 200 μl X-VIVO™ 15 (Lonza, Walkersville, MD, USA) supplemented with 20 ng/ml IL-2 for 48 h. The supernatants were subsequently collected and the level of IL-10 was determined using a commercial ELISA kit (MultiSciences, Hanzhou, China) according to the manufacturer's instructions.

### C1498 AML Model

This study was approved by the “Wenzhou Medical University Animal Care and Use Committee” and carried out in accordance with the recommendations of “institutional guidelines, Wenzhou Medical University Animal Care and Use Committee.” The C1498 mouse model of AML was established as described previously with minor modifications. Briefly, exponentially growing C1498 cells (5 × 10^6^) were resuspended in 100 μl PBS, and subsequently intravenously injected into the tail vein of recipient mice, which had been already exposed to 5 Gy myeloablative irradiation 4 h before. According to the methods previously reported (23), these mice were administrated intraperitoneally with 7.5 mg/kg anti-mouse PD-L1 antibody (clone: 10F.9G2) or with rat IgG2b isotype control (both from BioXcell, West Lebanon, NH, USA) on days 0, 3, 6, 9, 12, and sacrificed on day 15.

### Statistical Analysis

The data were presented as mean ± SEM and analyzed by *t*-tests or one-way ANOVA followed by a *post-hoc* Turkey's test to determine the differences between the groups. Differences at *P* < 0.05 were considered statistically significant. All statistical analyses were performed using Graphpad Prism 5.0 software.

## Results

### Expression and Induction of PD-L1 Molecules on AML Cells

**Figure 1:**
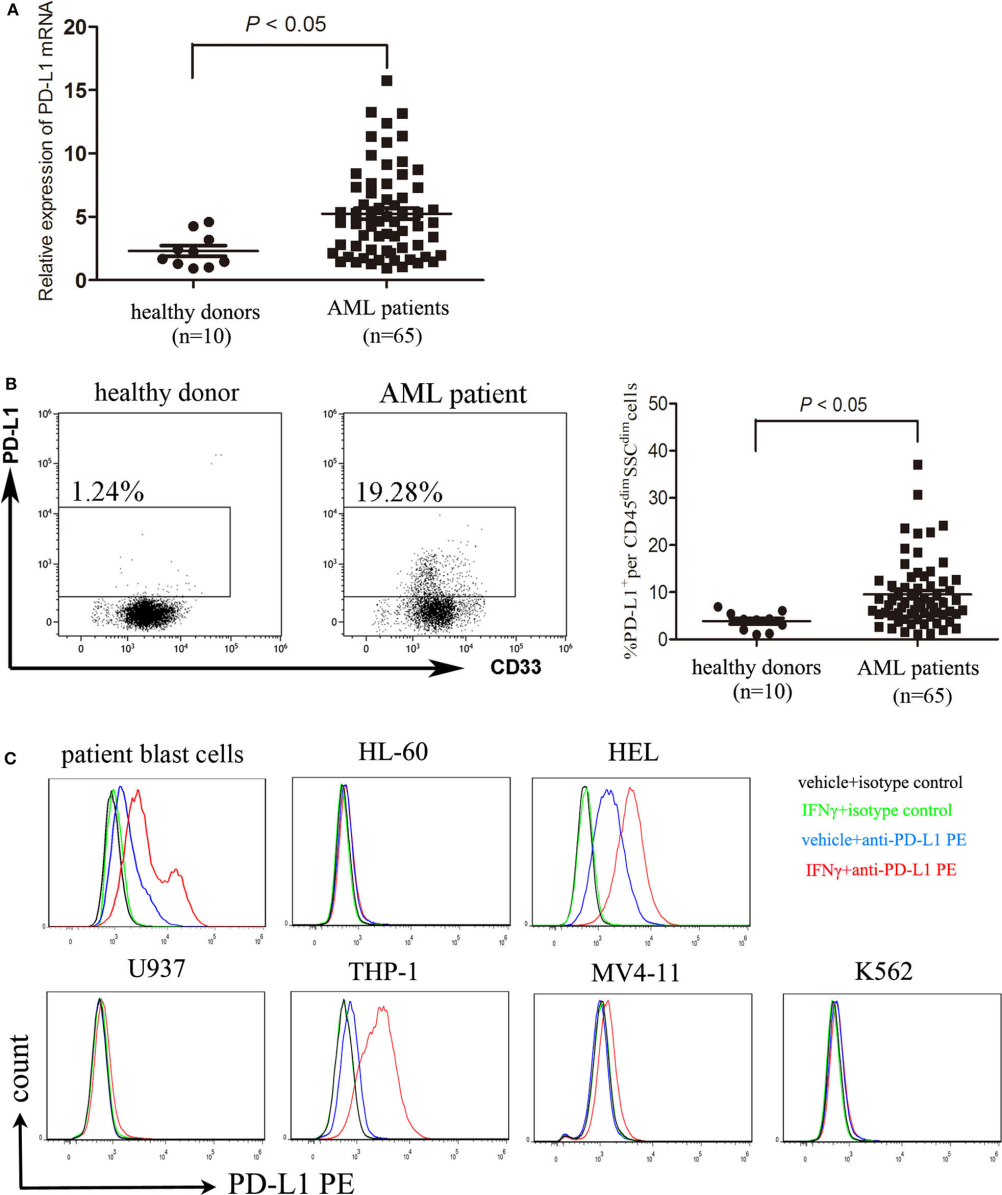
AML cells express PD-L1 and PD-L1 is upregulated by IFN-γ. **(A)** The mRNA expression of PD-L1 in BMMNCs isolated from 10 healthy donors and 65 patients with AML. **(B)** Representative dot plots (left panel) and statistical data (right panel) showing the expression of PD-L1 protein in CD45^dim^SSC^dim^ cells isolated from BM of 10 healthy donors and 65 patients with AML. Unpaired *t*-test was used to determine the difference. **(C)** Treatment with 400 U/ml IFN-γ for 48 h significantly upregulated the expression of PD-L1 in patient blast cells as well as two AML cell lines HEL and THP-1. Overlay histograms showing antibody stains with or without IFN-γ stimulation and isotype stains are representatives of three independent experiment.

It has been reported that the majority of human solid tumor cells express constitutively PD-L1 on the surface (24). The expression of PD-L1 protein on AML cells is controversial so far (13, 14). We showed that compared with BMMNCs isolated from healthy donors, blast cells from a substantial number of AML patients strongly expressed PD-L1 at the transcriptional level (Figure 1A). Though the expression of PD-L1 protein on patient blast cells of the majority of AML patients is rather weakly, it was higher in CD45^dim^SSC^+^ cells from AML patients than those from healthy donors (Figure 1B). Weak expression of PD-L1 protein were observed in six AML cell lines tested, and IFN-γ significantly upregulated the expression of PD-L1 in primary AML cells as well as two AML cell lines HEL and THP-1 *in vitro* (Figure 1C). However, IFN-γ 400 U/ml had little effect on the PD-L1 expression in other four AML cell lines tested (Figure 1C). The findings suggest that the upregulation of PD-L1 induced by IFN-γ stimulation may depend on cell of origin in AML, which significantly differs from the effect of IFN-γ on the vast majority of solid tumor cells (25, 26).

### BM-Infiltrating Treg Cells Express High Levels of PD-1 in Patients With AML

**Figure 2:**
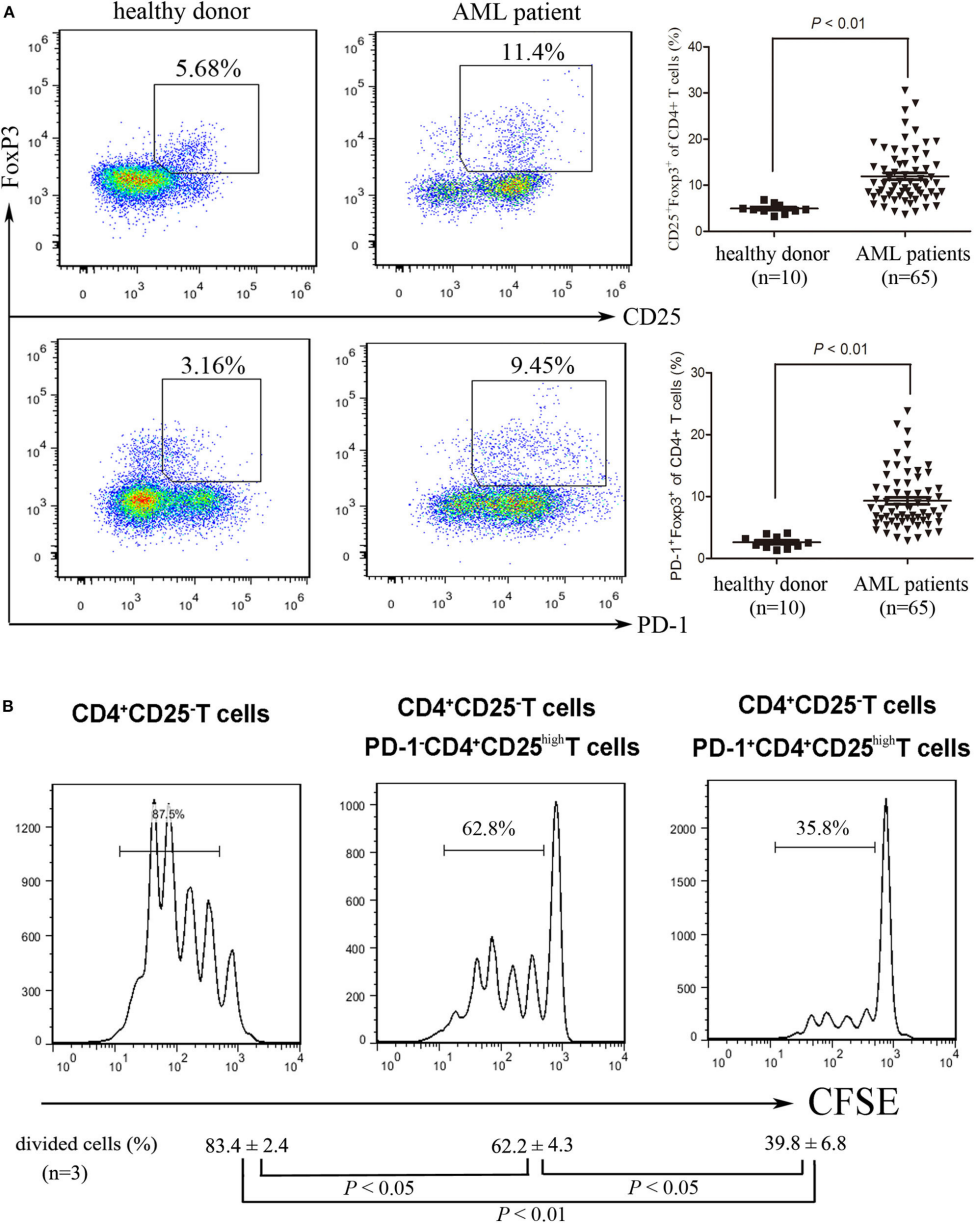
The frequency and function of PD-1^+^ Treg cells in patients with AML. **(A)** representative dot plots (left panel) and statistical data (right panel) showing the frequencies of Treg cells and PD-1^+^ Treg cells in BM isolated form healthy donors and patients with AML. Unpaired *t*-test was used to determine the difference. **(B)** CD4^+^CD25^high^PD-1^+^ cells and CD4^+^CD25^high^PD-1^−^ cells were isolated from BMMNCs of AML patients using flow cytometry, and then co-incubated with CFSE-labeled CD4^+^CD25^−^ T cells in the presence of PBMCs treated with 20 μg/ml mitomycin, with stimulation with plate-coated anti-CD3 (1 μg/ml) and soluble anti-CD28 (3 μg/ml) and IL-2 (20 ng/ml) for 5 days. CFSE Histograms were representatives of four independent experiments and ANOVA was used to determine the differences.

Although it is highly recognized that Treg cells are enriched in BM microenvironment in AML (5, 6), the detailed characteristics of these cells including the expression of immune inhibitory receptors remain poorly identified. We found that the frequency of BM-infiltrating Treg cells was higher in patients with AML than those in healthy donors (Figure 2A). Meanwhile, a similar result was also observed in the frequency of PD-1^+^ Treg cells (Figure 2A). The PD-1^+^Treg infiltration of the bone marrow had a trend to be associated with the PD-L1 expression on AML cells (*P* = 0.0548, Figure S1). We further investigated the inhibitory capability of the PD-1^+^CD4^+^CD25^high^ T cells against the conventional effector T cells. As shown in Figure 2B, PD-1^+^CD4^+^CD25^high^ T cells exhibited a greater inhibition of the proliferation of CFSE-labeled CD4^+^CD25^−^ T cells than the negative counterpart PD-1^−^CD4^+^CD25^high^ T cells from the same patients with AML, similarly to the results summarized by a previous report (27). In addition, we also found that PD-1 expression was up-regulated and IFN-γ production was decreased on CD8 cytotoxic T cells in bone marrow from patients with AML compared with those from healthy donors (Figure S2). Our data suggest that PD-1^+^Treg cells might be enriched in the BM microenvironment of patients with AML and exhibit a stronger inhibitory function than PD-1^−^ Treg cells.

### AML Cells Promote Treg Cell Expansion Through the Interaction of PD-1 and PD-L1

**Figure 3:**
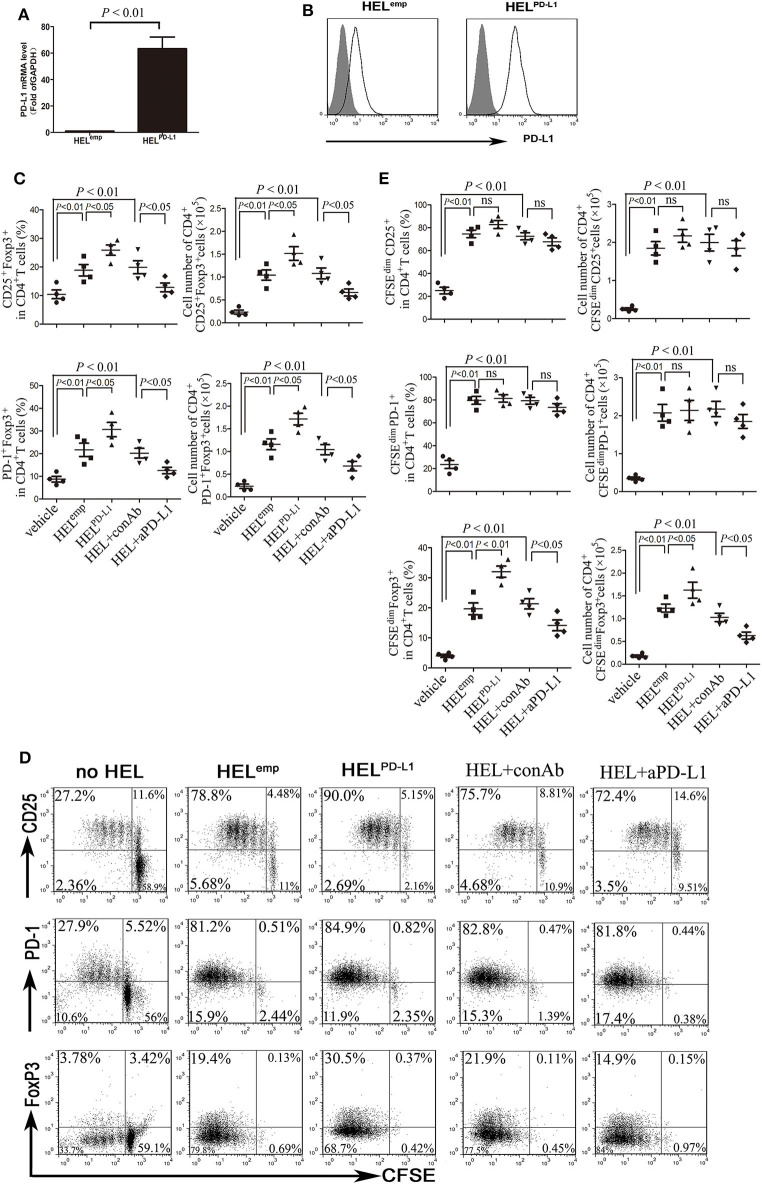
AML cells promote the expansion of Treg through PD-L1. **(A,B)** HEL cells were transduced to constitutively express full-length human PD-L1, and the mRNA and cell surface protein expression of PD-L1 were determined. Gray filled indicate the vehicle control, and black lines indicate the antibody staining. **(C)** Treg cells and PD-1^+^ Treg cells were significantly increased when CD4^+^ T cells co-cultured with HEL cells overexpressed PD-L1 for 5 days, and blockade of PD-1 signaling using anti-PD-L1 antibody markedly reduced the generation of Treg cells and PD-1^+^ Treg cells from CD4^+^ T cells. **(D,E)** HEL cells co-cultured with CFSE-labeled CD4^+^ T cells for 5 days, and subsequently determined the expansion of Treg cells and PD-1^+^ Treg cells. Representative images and statistical data of four independent experiments were shown.

To investigate whether PD-L1 contributes to the generation and expansion of Treg cells, we overexpressed PD-L1 in HEL cells by transducing plasmid carrying the full length human PD-L1 gene (Figures 3A,B). HEL cells with forced expression of PD-L1 have a stronger induction of Treg cells from CD4^+^ T cells than those transduced with NC plasmids (Figure 3C). More importantly, the frequency of PD-1^+^Treg cells induced by HEL cells overexpressed PD-L1 was also higher than those induced by HEL cells transduced with NC plasmids (Figure 3C). Meanwhile, a neutralizing anti-PD-L1 antibody was used to block the interaction of PD-1 and PD-L1, which dramatically inhibited the induction of Treg cells from CD4^+^ T cells, especially PD-1^+^ Treg cells (Figure 3C). We also analyzed the effect of HEL cells on the expansion of CD4^+^ T cells using the intracellular fluorescent dye CFSE. Co-culture with HEL cells led to the majority of conventional CD4^+^ T cells being activated and activation-induced anergy (Figures 3D,E). Overexpression of PD-L1 strongly promoted the expansion of FoxP3^+^ Treg cells and blockade of PD-1 signaling by anti-PD-L1 antibody drastically inhibited the expansion of Treg cells (Figures 3D,E). Meanwhile, anti-PD-L1 antibody attenuated Th1-type cytokine IFN-γ production and Th17-type cytokine IL-17 production (Figure S3).

### IL-10 and IL-35 Secreted by Treg Cells Promote the Proliferation of AML Cells

**Figure 4:**
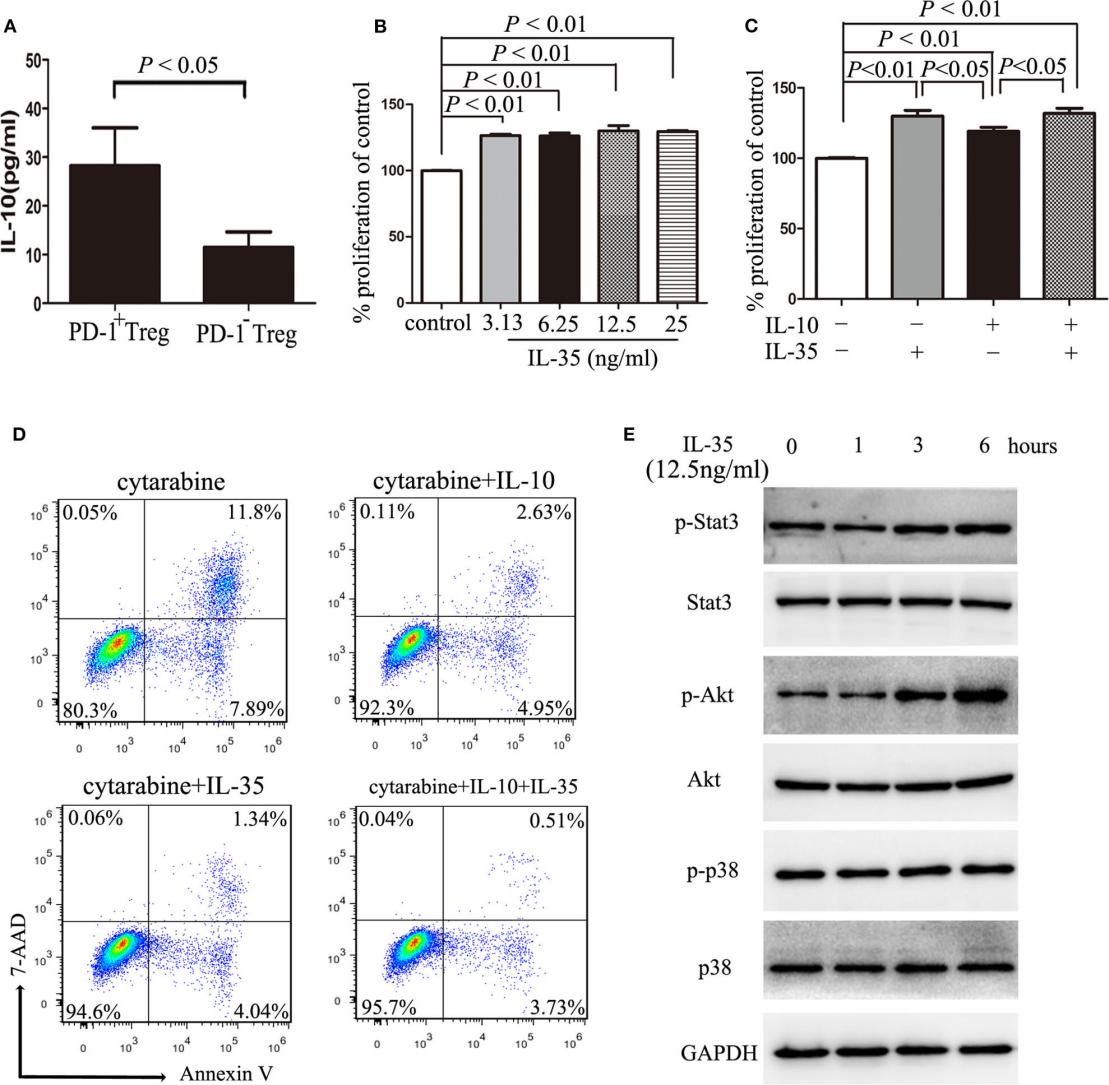
IL-10 or IL-35 promotes the proliferation of AML cells. **(A)** The CD4^+^CD25^high^PD-1^+^ cells and CD4^+^CD25^high^PD-1^−^ cells were sorted and cultured in a total volume of 200 μl X-VIVO™ 15 supplemented by 20 ng/ml IL-2 for 48 h, and then, the level of IL-10 in the cell supernatants was determined by ELISA. Results were expressed as the mean ± SEM of four independent experiments, and unpaired *t*-test was used to determine the difference. **(B)** Treatment with IL-35 more than 3.13 ng/ml for 48 h promoted the proliferation of HL-60 cells. **(C)** Treatment with IL-10 10 ng/ml for 48 h had no synergistic effects on the proliferation of HL-60 cells with IL-35 12.5 ng/ml. **(D)** Treatment with IL-10 10 ng/ml or IL-35 12.5 ng/ml for 24 h reduced cytarabine-induced apoptosis of HL-60 cells. **(E)** IL-35 upregulated the phosphorylation of Akt but not Stat3 or p38 within 6 h after stimulation in HL-60 cells. Representative images and statistical data of four independent experiments were shown.

Our previous study have found that IL-10, one cytokine secreted by Treg cells, can promote the expansion of AML cells (6). Interestingly, PD-1^+^ Tregs produced markedly more IL-10 than PD-1^−^ Tregs (Figure 4A). Similarly to IL-10, IL-35, another classical cytokine secreted by Treg cells (28, 29), also promoted the proliferation of HL-60 cells *in vitro* (Figure 4B). Regrettably, IL-35 and IL-10 had no synergistic effect on the proliferation of HL-60 cells (Figure 4C). IL-35 or IL-10 alone reduced drug-induced apoptosis by cytarabine *in vitro*, but these two cytokines had no synergistic effects (Figure 4D). Additionally, IL-35 significantly upregulated the phosphorylation of Akt but not Stat3 or p38 within 6 h after stimulation (Figure 4E), suggesting that the activation of PI3K/Akt signaling pathway may be one of the main mechanism of IL-35 promoting the proliferation of AML cells.

### Blockade of PD-L1 Reduces the Frequency of Treg Cells and Tumor Burden in the C1498 Mouse Model

**Figure 5:**
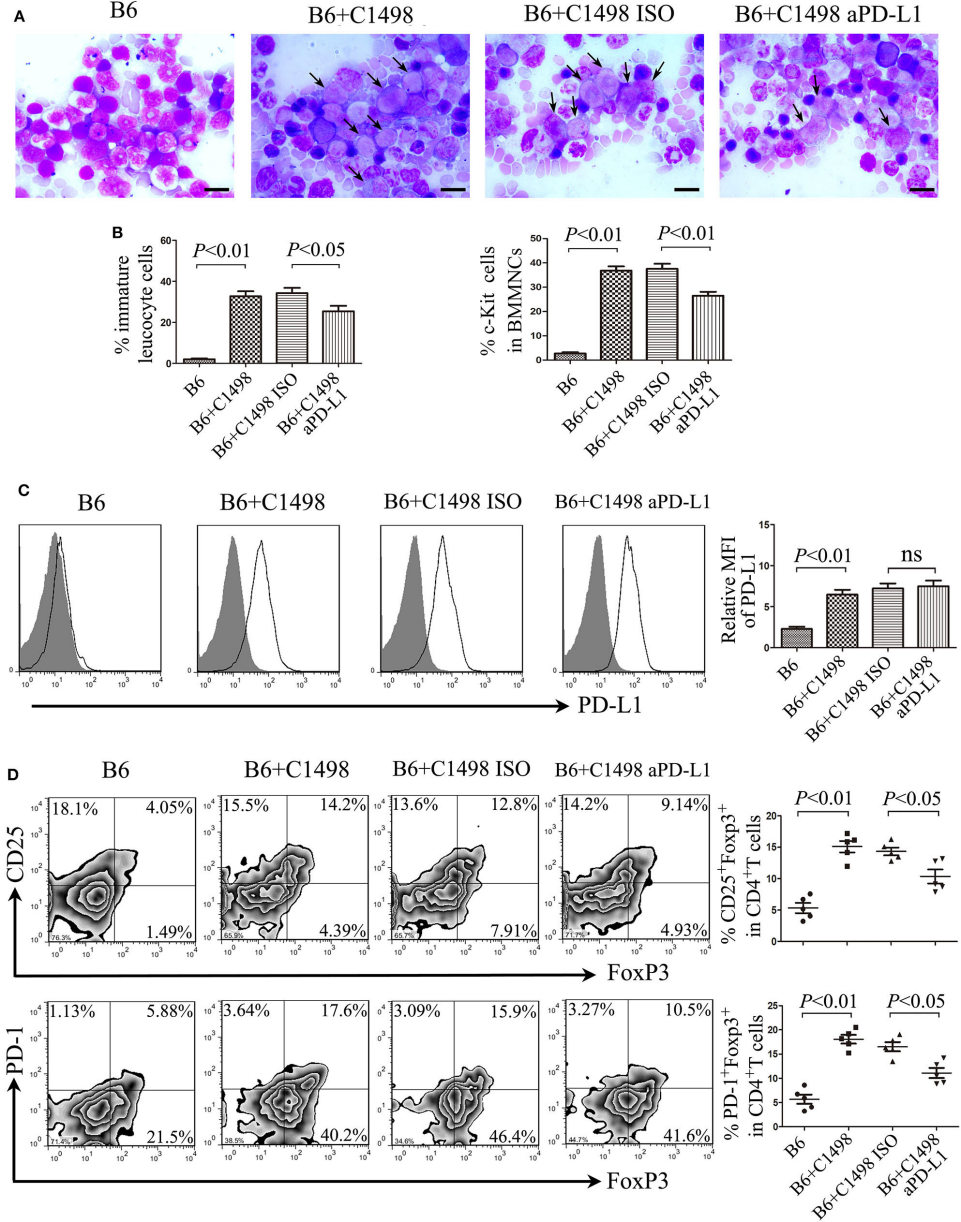
Anti-PD-L1 antibody delays tumor progression of the C1498 mouse model by impairing the expansion of Treg cells. **(A)** BM smears were stained with Wright's stain, and representative images and statistical data of immature leucocyte cells were shown. The scale bars equal 10 μm, and arrows indicate immature leucocyte cells. **(B)** Statistical data of the expression of c-Kit in BMMNCs were shown. **(C)** Representative overlay histograms and statistical data of the expression of PD-L1 of BMMNCs were shown. Gray-filled indicate isotype-matched control, and black solid lines indicate antibody staining. **(D)** The frequencies of Treg cells and PD-1^+^ Treg cells were dramatically increased in the BM microenvironment of C1498-injected mice, and blockade of PD-1 signaling by anti-PD-L1 antibody attenuated drastically the expansion of Treg cells and PD-1^+^ Treg cells in BM. Images shown were representatives of 5 individual mice each group, and statistical data were analyzed using ANOVA. NS stands for not significant.

On 15 days after intravenous injection of C1498 cells, the mouse model of AML had been established according to BM examination, and antibody blockade of PD-L1 moderately reduced tumor burden indicated by Wright's staining and flow cytometric analysis of c-Kit staining (Figures 5A,B; Figure S4). Meanwhile, the expression of PD-L1 in mononuclear cells isolated from BM (Figure 5C), PB and spleen (Figure S5A) was drastically upregulated. As expected, blockade of PD-L1 did not affect the expression of PD-L1 on mononuclear cells from these tissues (Figure S5A). Importantly, not only the frequency of total Treg cells, but also the frequency of PD-1^+^ Treg cells were increased dramatically in BM (Figure 5D), PB, and spleen (Figures S5B,C) in C1498 mouse model compared to those of the vehicle mice. In addition, reduced Th1 cell frequency and elevated Th17 frequency were also emerged in BM and spleen from C1498 mouse model compared to those from the vehicle mice (Figure S6). Antibody blockade of PD-L1 significantly attenuated the frequencies of Treg cells and PD-1^+^ Treg cells in BM (Figure 5D), PB, and spleen (Figures S5B,C). As expected, blockade of PD-L1 also changed the immune status with a tremendous increase of the frequency of Th1 cells and a dramatic decrease of the frequency of Th17 cells (Figure S6). These data reveal a critical role of PD-1/PD-L1 signaling for the expansion of PD-1^+^ Treg cells.

### Frequencies of Treg Cells and PD-1^+^ Treg Cells Correlate With Patient Prognosis in AML

**Figure 6:**
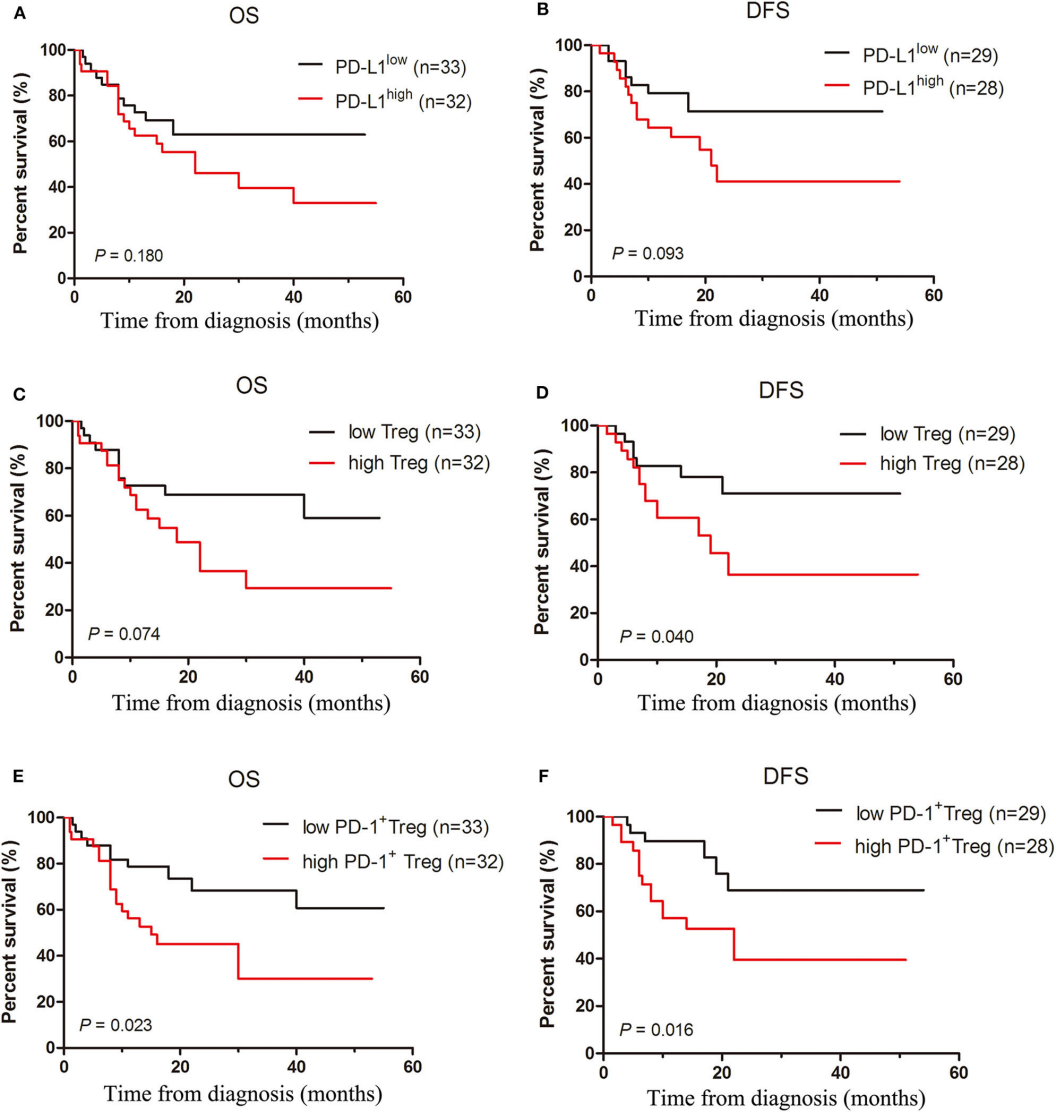
Increased frequencies of Treg cells and PD-1^+^Treg cells rather than the expression of PD-L1 predict poor survival in AML patients. Overall and disease-free survival curves were estimated for PD-L1 expression of patient AML cells **(A,B)**, frequencies of Treg cells **(C,D)** and PD-1^+^ Treg cells **(E,F)** in BM microenvironment of 65 patients with AML using the Kaplan-Meier method and differences in survival distributions were evaluated by the log-rank test.

Mounting evidences have demonstrated that circulating or tumor-infiltrating Treg cell frequency may serve as independent prognostic factors in many types of tumor and Treg cells are alternatively harmful or beneficial to patient survival. As PD-L1 can induce the generation and expansion of Treg cells, as well as restrict effector T cell function and expansion, we analyzed whether the PD-1/PD-L1 pathway involved in regulating T-cell responses correlates with patient outcome. We classified these cases into two groups according to the median of PD-L1 positivity in patient AML cells and found that these two groups had no statistical difference in overall survival (OS) and DFS (Figures 6A,B). When AML patients were classified into two groups using the median frequency of Treg cells in BM, cases in high Treg cell frequency showed a markedly shorter DFS and a short but not statistically significant OS compared with those in low Treg cell frequency group (Figures 6C,D). Meanwhile, the relation of the frequency of PD-1^+^ Treg cells and patient survival was also analyzed. The OS and DFS in high PD-1^+^ Treg cell group were shorter than those in low PD-1^+^ Treg cell group (Figures 6E,F). In addition, these patients were also stratified by risk according to ELN classification (30). We carried out a multivariate Cox regression analysis that included risk stratification, PD-L1 positivity, frequency of Treg, and frequency of PD-1^+^Treg, and found that risk stratification had statistical significance to OS and had a slight effect on DFS, but three indictors including PD-L1 positivity, frequency of Treg and frequency of PD-1^+^Treg are not independent prognostic predictors to OS and DFS in this multivariate analysis (Table S2).

## Discussion

Cancer progression is a multi-step process that depends on both tumor behavior and the host immune system. The immune system in most patients with cancer is generally compromised (31). The intratumoral accumulation of Treg cells has been associated with high tumor burden and metastasis and reduced survival in several mouse tumor models, and, more importantly, with advanced-stage disease and poor prognosis in patients with cancer (32). Furthermore, elevated Treg cell number in tumor microenvironment is also observed and these PD-1^+^ Treg cells are markedly associated with cancer development and progression (33). In our present study, we revealed a higher frequency of Treg cells in the BM from patients with AML compared with those from healthy donors, similar to those previous studies that showed obvious infiltration of regulatory lymphocytes into the tumor tissue in some types of solid tumors and hematological malignancies (34, 35). We also showed that IFN-γ could upregulate the expression of PD-L1 on AML cells. Blockade of PD-1 signaling by anti-PD-L1 antibody impaired the interaction between AML cells and Treg cells and improved diseases.

It is generally recognized that PD-L1 is extensively detectable on the majority of tumors including solid and hematological malignancies (36, 37). In AML, the PD-L1 overexpression usually occurred during therapy, after alloHSCT (38) and therapy with hypomethylating agents (39), such as azaticidine and decitabine, and at the relapse of the disease. In this study, we have found that PD-L1 is expressed variably on several AML cell lines and patient blast cells. IFN-γ was capable of enhancing the expression of PD-L1 on patient blast cells as well as AML cell lines THP-1 and HEL *in vitro*. We also showed that PD-L1 expressed by AML cells could induce the expansion of Treg cells with high levels of FoxP3, CD25, and PD-1. In this process, AML cells themselves may be acted as direct APC. Meanwhile, PD-L1 blockade led to decreased expression of FoxP3 but did not eradicate it, suggesting that some other factor may also affect the FoxP3 expression. It has been reported that some molecules expressed by AML cells, such as IDO (9) and ICOSL (6), can promote the expansion of Treg cells in the tumor microenvironment.

Chronic exposure to antigen can lead to exhaustion of antigen-specific effector CD8^+^ T cells occurring in some infections and most cancers (40). Exhausted T cells acquires a dysfunctional state characterized by the expression of inhibitory receptors including PD-1. However, the expression of PD-1 on inhibitory immune cells including Treg cells may has a distinct effect for their functions. In our present study, PD-1^+^ Treg cells produced more IL-10, a main cytokines secreted by Treg cells, than PD-1^−^ Treg cells. Meanwhile, PD-1^+^ Treg cells have a stronger suppressor function than PD-1^−^ Treg cells. Above two features suggest that PD-1^+^ Treg cells are not exhausted T cells. Therefore, we think that PD-1^+^ Treg cells are potent, but not exhausted, conflicting with some previous reports (41).

IL-35 is mainly produced by Treg cells and regulatory B cells and plays a central role in the development and prevention of infectious, autoimmune and malignant diseases (42, 43). IL-35 as an inhibitory cytokine is capable of suppressing the proliferation of T cells and promoting the conversion of conventional T cells into IL-35-producing induced Treg cells and downregulating Th17 cell development and differentiation (44, 45). In this study, we found that similar to IL-10, IL-35 had also a proliferation promotion on AML cells. Regrettably, IL-10 and IL-35 have no a synergistic effect on proliferation promotion on AML cells. The induction of PD-1^+^ Treg cells further raised the possibility of more IL-10 and IL-35 accumulation in tumor microenvironment, which orchestrated a positive feedback loop contributing to AML cell proliferation. In addition, it has been shown that IL-35 secreted by breast cancer cells also promotes the IL-10 production and sharply decreases the secretion of Th1-type cytokine IFN-γ and IL-17 in conventional T cells (28). In this study, we also showed that IL-35 or IL-10 protected AML cells from cytarabine-induced apoptosis. Our results suggest that IL-35 may promote tumor progression through inducing the expansion of AML cells.

Increased PD-1 expression on Treg cells resulting in stronger inhibition of the immune response may contribute to the progression of AML (46). In this study, in mice injected with a murine AML cell line C1498, tumor progression led to increased Treg cells and elevation of PD-1 expression on Treg cells in the BM microenvironment. Blockade of PD-1 signaling by anti-PD-L1 antibody reduced leukemia cell burden in AML mice, at least partly by inhibiting the expansion of PD-1^+^ Treg cells and reversing inhibitory cytokine production. The finding may have important clinical consequences. If the immunosuppression can be overcome, it is possible that the residual blasts can be killed by the patient's own immune system or a graft-vs-leukemia effect. Therapy that aims at this immunosuppressive target of AML could be given as a maintenance treatment after consolidation chemotherapy or after bone marrow transplantation. Several phases I/II clinical trials for checkpoint inhibitors in monotherapy or combined treatment for AML have started in the last few years, and the patients can be benefit from some clinical trials according to the premilinary results (46, 47).

**Figure 7:**
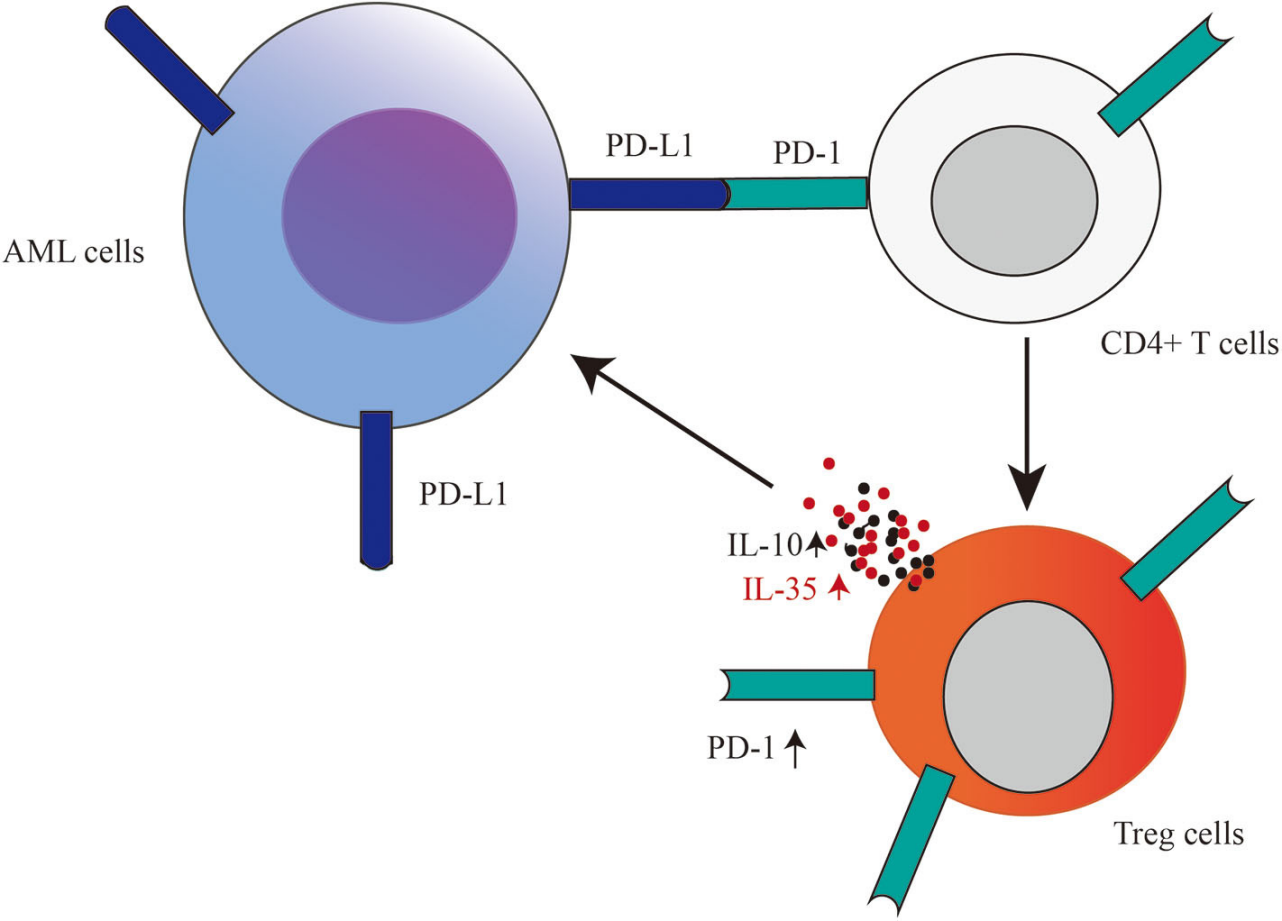
Based on our results, we propose that PD-L1 expressed in AML cells may potentiate Treg cell expansion, and Treg cell producing-IL-35 or IL-10 as a positive feedback may further promote the proliferation of AML cells.

Thus, we propose that PD-L1 expressed in AML cells may potentiate Treg cell expansion, and IL-35 or IL-10 produced by Treg cells may prompt the proliferation of AML cells (Figure 7). Further studies are required to confirm and dissect the detailed mechanisms. Our present data, including study of PD-L1 blockade in leukemic mice, suggest that anti-PD-L1 antibody administration may be a promising therapeutic option to inhibit Treg cell-induced immunosuppression, especially in some settings, such as after alloHSCT and therapy with hypomethylating agents and at the relapse of this disease.

## Data Availability Statement

All datasets generated for this study are included in the article/Supplementary Material.

## Ethics Statement

This study received approval from the Institutional Ethics Committee of the First Affiliated Hospital of Wenzhou Medical University, and all participants signed written informed consent in accordance with the Declaration of Helsinki.

## Author Contributions

SZ and KY designed the study and supervised the manuscript preparation. YD, YHa, YHu, and SZ performed the experiments. YD, YHa, YHu, SJ, and SZ analyzed the data. SZ wrote the manuscript. ZH, RC, and ZY participated in the collection of patients' data. All of the authors agreed to submit the final manuscript.

## Conflict of Interest

The authors declare that the research was conducted in the absence of any commercial or financial relationships that could be construed as a potential conflict of interest.
