# Supplementary material for: PD-L1 Is Expressed and Promotes the Expansion of Regulatory T Cells in Acute Myeloid Leukemia
Source: Front Immunol. 2020 Jul 31;11:1710. doi: 10.3389/fimmu.2020.01710 (PMC7412746; doi:10.3389/fimmu.2020.01710)
Supplement: Supplementary file 1 [file Data_Sheet_1.docx]

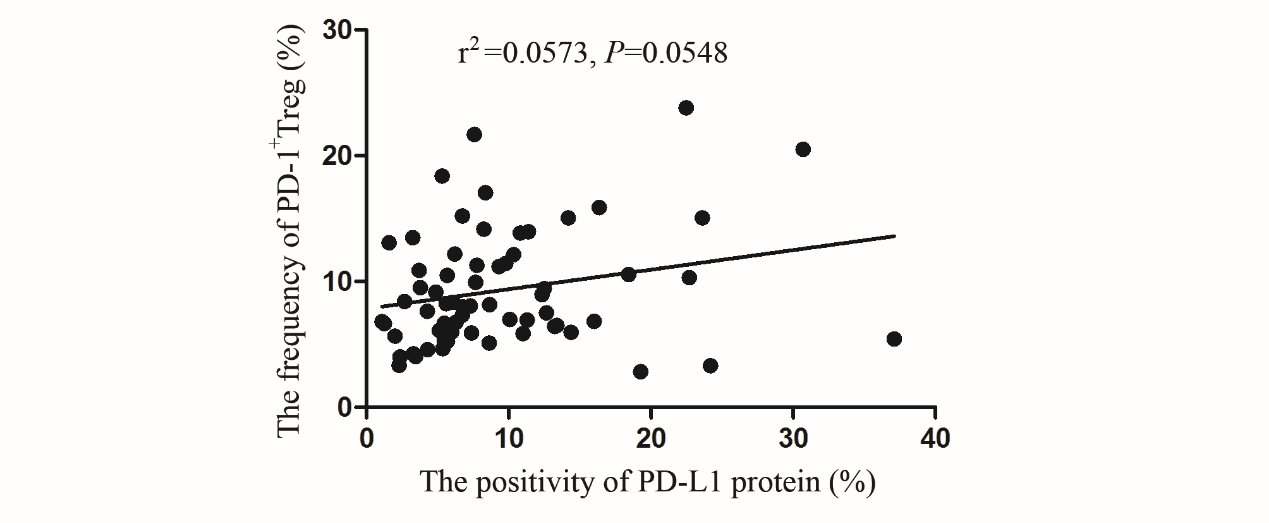


**Figure S1** The positivity of PD-L1 protein in patient blast cells has a trend to be associated with the frequency of bone marrow-infiltrating PD-1^+^ Treg in patients with AML using Pearson correlation analysis.


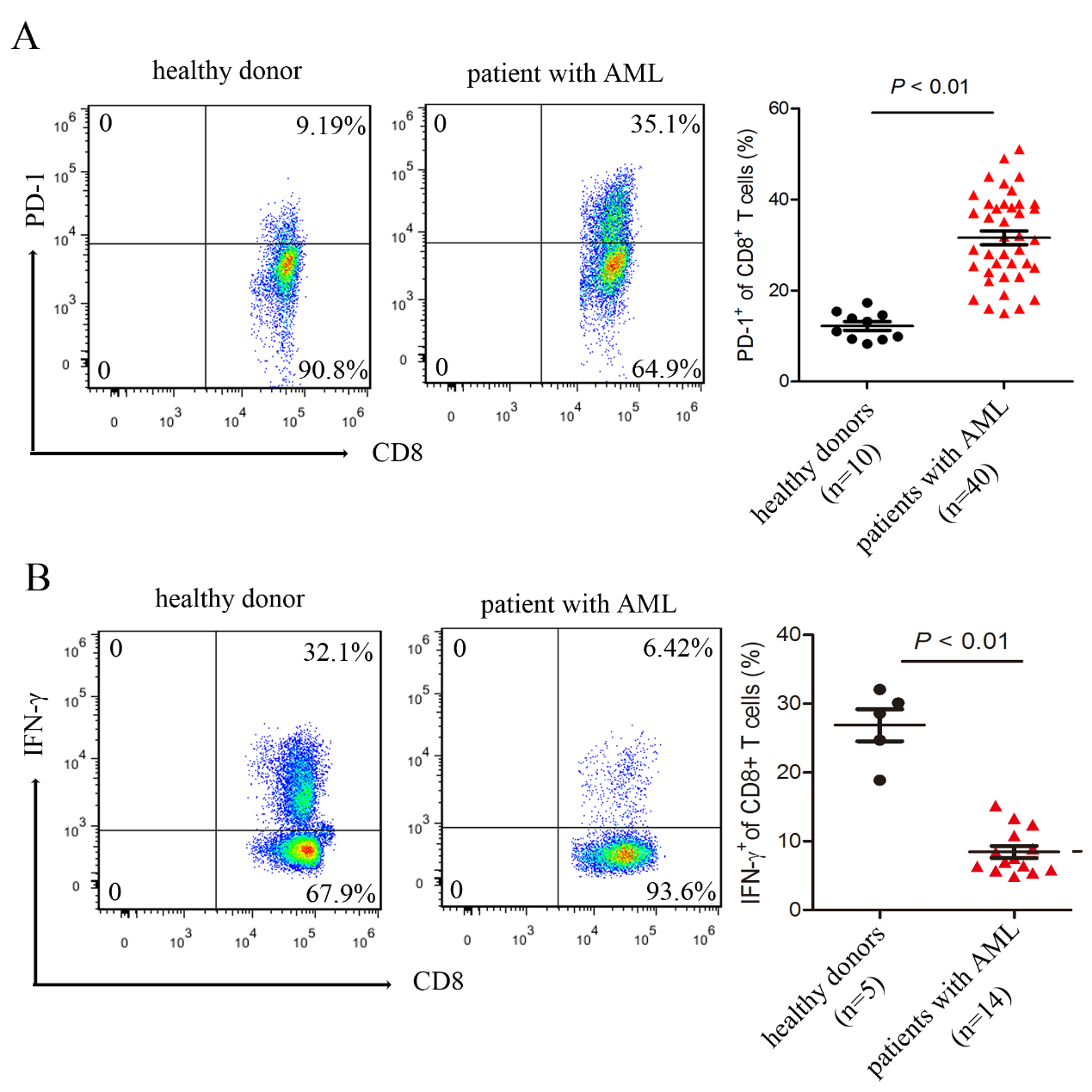


**Figure S2** The expression of PD-1 was up-regulated and the production of IFN-γ was reduced in bone marrow infiltrating CD8^+^ T cells in patients with AML compared than those from healthy donors. (**A**) Respective dot plots and statistical data of the expression of PD-1 in bone marrow infiltrating CD8^+^ T cells in patients with AML and healthy donors. (**B**) Respective dot plots and statistical data of the IFN-γ production in bone marrow infiltrating CD8^+^ T cells in patients with AML and healthy donors.


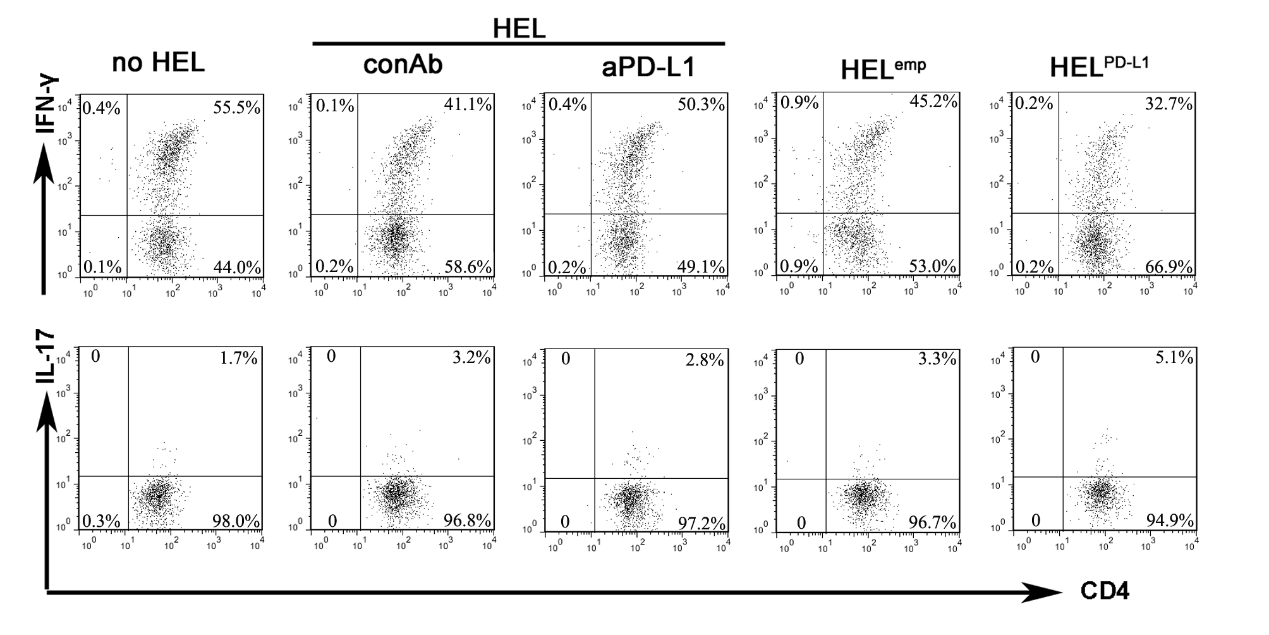


**Figure S3** The effect of PD-L1 expression of AML cells on the induction of Th1 and Th17 cells from CD4^+^ T cells. CD4^+^ T cells were co-cultured with HEL cells with or without anti-PD-L1 antibody for 48 h, or with HEL cells overexpressed PD-L1 or vehicle control for 48 h. The frequencies of IFN-γ-producing (Th1) cells and IL-17A-producing (Th17) cells were determined using a flow-cytometry-based assay. Representative images of three independent experiments were showed.


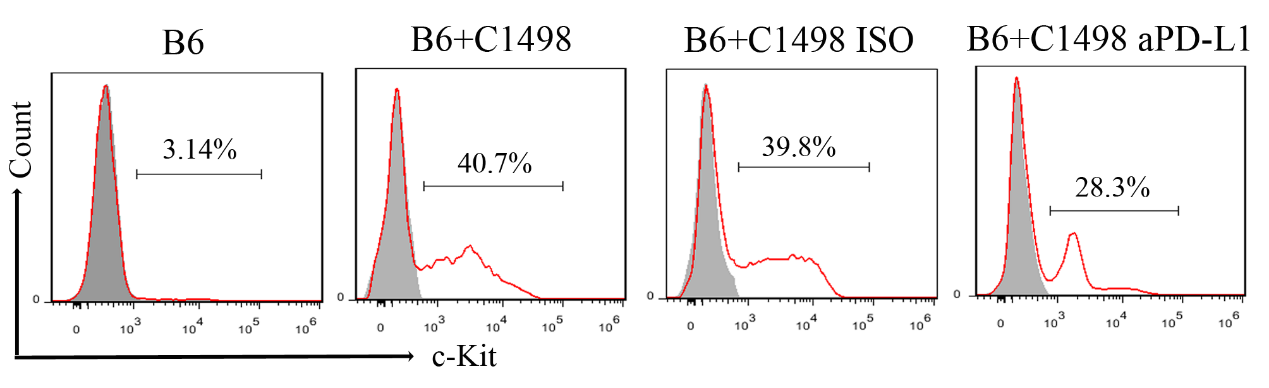


**Figure S4** Representing histograms of the expression of c-Kit in BMMNCs were shown. Gray-filled indicate isotype-matched control, and red solid lines indicate antibody staining.


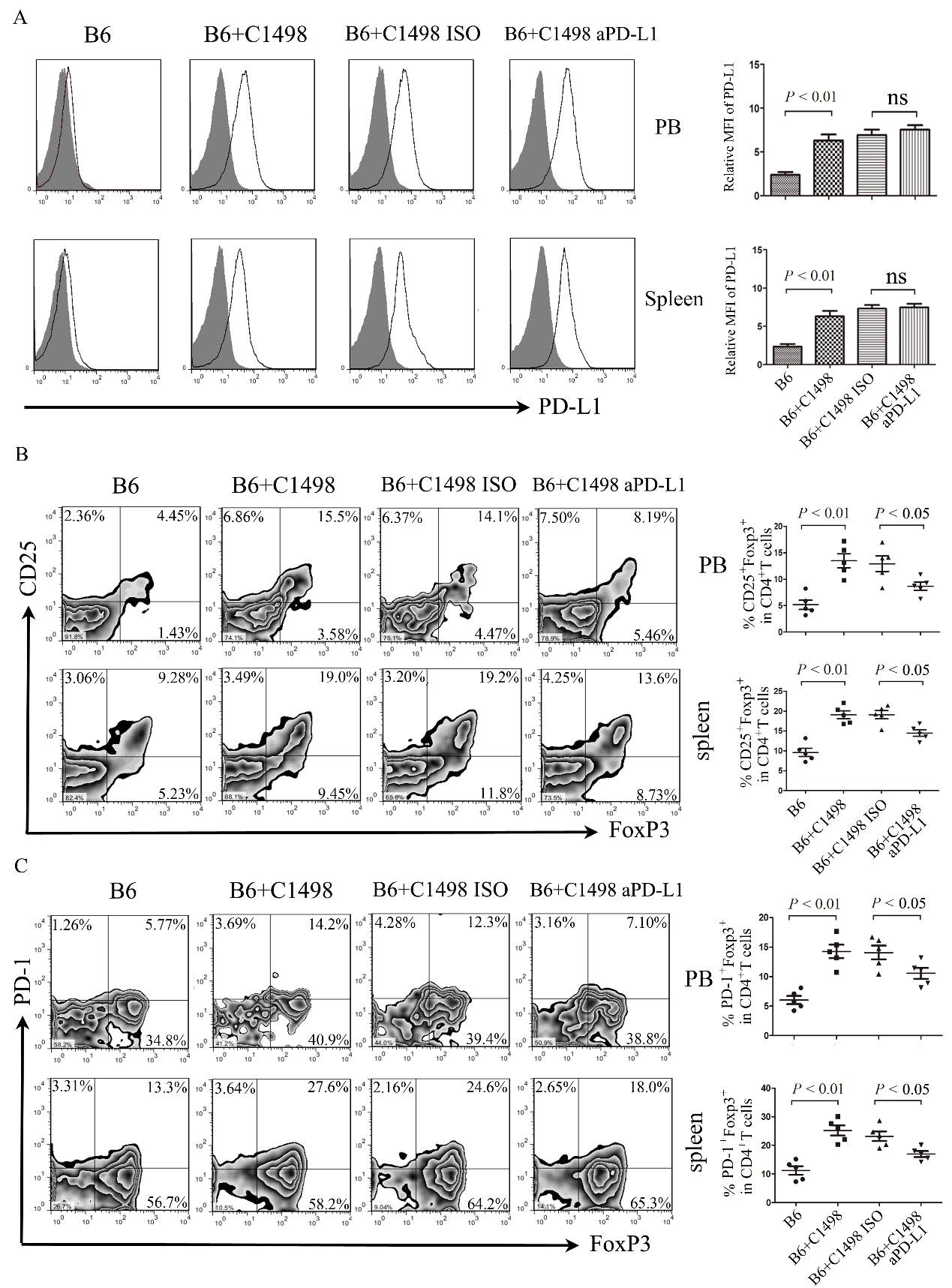


**Figure S5** Blockade of PD-1 signaling by anti-PD-L1 antibody impairs the expansion of Treg cells in PB and spleen in C1498-injected mice. **(A)** overlay histograms showing isotype-matched control (filled gray) and antibody staining (solid black) represented the expression of PD-L1 of PB mononuclear cells and spleen cells. **(B, C)** The frequencies of Treg cells and PD-1^+^ Treg cells were increased enormously in the PB and spleen of C1498-injected mice and anti-PD-L1 antibody drastically reduced the expansion of Treg cells and PD-1^+^ Treg cells in PB and spleen. Images shown were representatives of 5 individual mice each group, and statistical data were analyzed using ANOVA. NS stands for not significant.


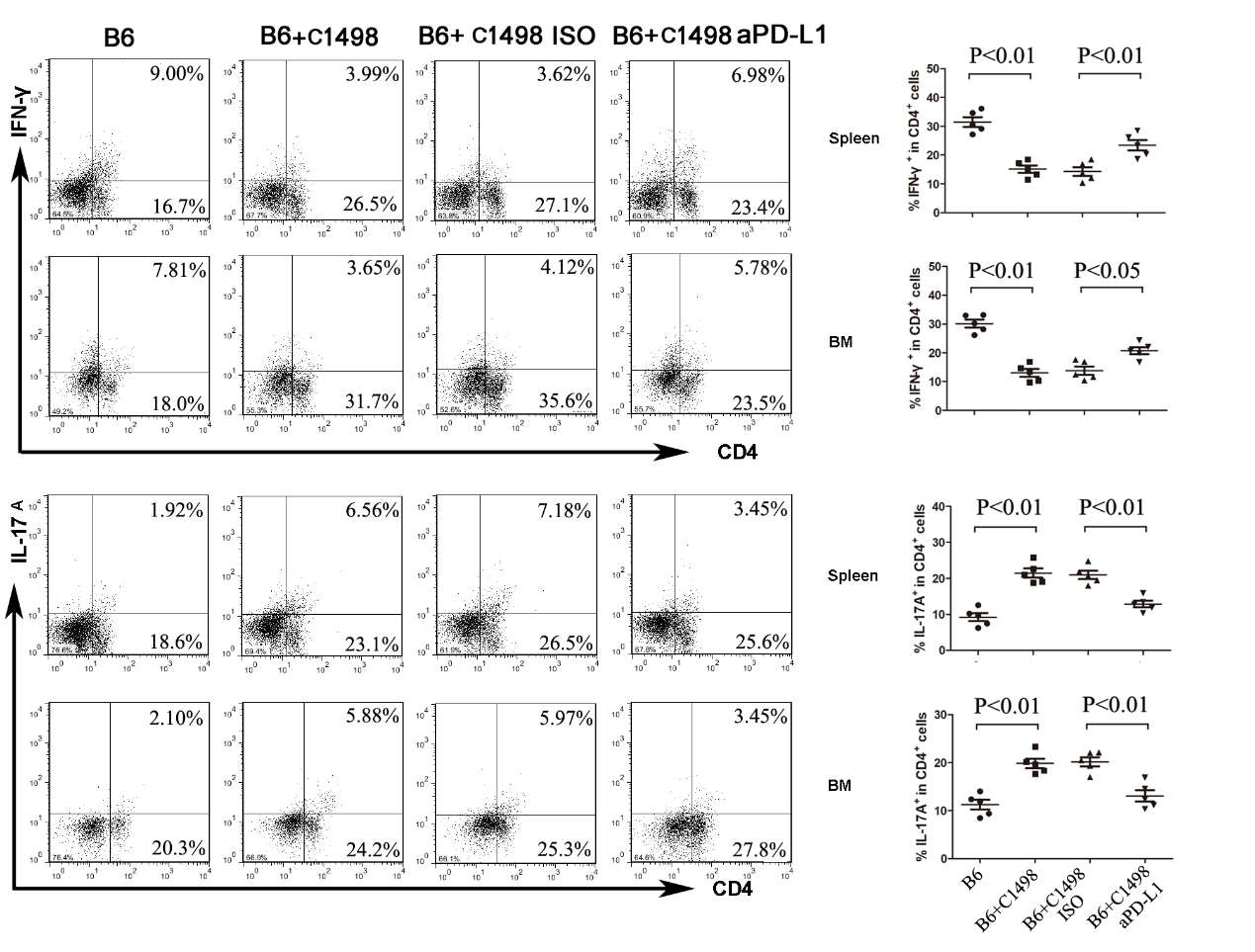


**Figure S6** Blockade of PD-1 signaling by anti-PD-L1 antibody increases Th1 cells and decreases Th17 cells in C1498-injected mice. After treatment with anti-PD-L1 antibody, the frequencies of Th1 and Th17 cells in BM and spleen were determined. Representative images (left panel) showing IFN-γ-producing (Th1) cells and IL-17A-producing (Th17) cells and statistical data (right panel) were analyzed using ANOVA. NS stands for not significant.

**Table S1. Clinical characteristics of patients with non-M3 acute myeloid leukemia.**

| **Patient no** | **Sex** | | **Age, y** | **2016 WHO Classification** | **Cytogenetic abnormalities** | **Molecular aberrations** | **Included date** |
| --- | --- | --- | --- | --- | --- | --- | --- |
| 1 | M | 34 | | Acute myelomonocytic leukemia | 46, XY | —* | 2014/12/26 |
| 2 | M | 45 | | Acute monoblastic/monocytic leukemia | 46, XY | — | 2015/01/20 |
| 3 | M | 20 | | AML with t(16;16)(p13.1;q22); CBFB-MYH11 | 46, XY, t(16;16)(p13.1;q22) | CBFB-MYH11+ | 2015/01/26 |
| 4 | M | 23 | | Acute monoblastic/monocytic leukemia | 46, XY | WT1+ | 2015/03/13 |
| 5 | M | 17 | | AML with t(8;21)(q22;q22); RUNX1-RUNX1T1 | 46, XY, t(8;21)(q22;q22) | AML1-ETO+ | 2015/03/17 |
| 6 | M | 37 | | Acute myelomonocytic leukemia | 46, XY | — | 2015/03/19 |
| 7 | F | 56 | | Acute myelomonocytic leukemia | 46, XX | WT1+ | 2015/04/07 |
| 8 | F | 46 | | Acute myelomonocytic leukemia | 46, XX | — | 2015/04/08 |
| 9 | M | 49 | | Acute monoblastic/monocytic leukemia | 46, XY | WT1+ | 2015/04/24 |
| 10 | F | 25 | | Acute monoblastic/monocytic leukemia | 46, XX | WT1+ | 2015/06/02 |
| 11 | M | 52 | | AML with t(8;21)(q22;q22); RUNX1-RUNX1T1 | 45, X, -Y, t(8;21;20)(q22;q22;p12) | AML1-ETO+ | 2015/07/10 |
| 12 | M | 60 | | AML with maturation | 40-42, XY, inv(3)(p21p13), del(4) (q25q31), del(15)(q13q33), der(6), del(7)(q22q34), add(p15) | — | 2015/07/14 |
| 13 | F | 37 | | AML with mutated CEBPA | 46, XX, del(9)(p13q22) | CEBPA double-mut | 2015/09/17 |
| 14 | M | 24 | | AML with t(16;16)(p13.1;q22); CBFB-MYH11 | 46, XY, t(16;16)(p13.1;q22) | CBFB-MYH11+ | 2015/09/24 |
| 15 | M | 46 | | AML with t(16;16)(p13.1;q22); CBFB-MYH11 | 46, XY, inv(16)(p13q22) | CBFB-MYH11+ | 2015/10/30 |
| 16 | M | 39 | | AML with mutated CEBPA | 46, XY | CEBPA double-mut | 2015/11/02 |
| 17 | F | 55 | | AML with maturation | 46, XX | — | 2015/11/02 |
| 18 | M | 47 | | AML with maturation | 46, XY, inv(9)(p12q13) | CBFB-MYH11- | 2015/11/10 |
| 19 | F | 41 | | AML with maturation | 92, XXXX | — | 2015/11/19 |
| 20 | F | 55 | | Acute monoblastic/monocytic leukemia | 46, XX | FLT3-ITD+ | 2015/12/07 |
| 21 | F | 70 | | Acute myelomonocytic leukemia | 46, XX | — | 2017/04/08 |
| 22 | F | 21 | | AML with t(16;16)(p13.1;q22); CBFB-MYH11 | 46, XX, inv(16)(p13q22) | CBFB-MYH11+ | 2017/04/14 |
| 23 | F | 34 | | AML without maturation | 46, XX, del(7)(q31) | — | 2017/04/17 |
| 24 | F | 23 | | AML with maturation | 46, XX | — | 2017/06/16 |
| 25 | M | 57 | | AML with maturation | 46, XY, del(7)(q22q34) | — | 2017/06/26 |
| 26 | F | 49 | | Acute monoblastic/monocytic leukemia | 46, XX | — | 2017/07/15 |
| 27 | F | 65 | | Acute monoblastic/monocytic leukemia | 46, XX | FLT3-ITD+ | 2017/11/01 |
| 28 | F | 18 | | Acute monoblastic/monocytic leukemia | 46, XX | — | 2017/11/13 |
| 29 | M | 72 | | AML with t(8;21)(q22;q22); RUNX1-RUNX1T1 | 45, X, -Y, t(8;21)(q22;q22) | AML1-ETO+ | 2017/11/14 |
| 30 | M | 39 | | Acute monoblastic/monocytic leukemia | 46, XY | — | 2017/11/15 |
| 31 | F | 50 | | Acute monoblastic/monocytic leukemia | 46, XX | — | 2017/11/23 |
| 32 | F | 30 | | Acute monoblastic/monocytic leukemia | 47, XX, der(5), t(3;5)(q21;q31), del(5) (q12;q13), del(9)(q13;q22), del(16) t(16;18)(q24;q23), dup(16) (p11.2;q13.1), add(18)(q11.2) | — | 2017/12/11 |
| 33 | M | 45 | | AML with maturation | 46, XY, t(2;11)(p21;q23) | — | 2017/12/21 |
| 34 | F | 30 | | Acute monoblastic/monocytic leukemia | 46, XX | — | 2018/01/26 |
| 35 | M | 76 | | Acute monoblastic/monocytic leukemia | 46, XY | — | 2018/03/30 |
| 36 | F | 66 | | AML with mutated NPM1 | 46, XX | NPM1 mut | 2018/04/02 |
| 37 | M | 27 | | Acute monoblastic/monocytic leukemia | 46, XY | — | 2018/05/07 |
| 38 | F | 62 | | AML with maturation | 46, XX | — | 2018/05/24 |
| 39 | F | 62 | | Acute monoblastic/monocytic leukemia | 47, XX, t(6;13)(p21;q30), +11 | TET2 50%, 22.47% double-mut; FLT3-ITD ins 70 bp | 2018/05/28 |
| 40 | F | 50 | | Acute monoblastic/monocytic leukemia | 46, XX | — | 2018/06/04 |
| 41 | F | 54 | | AML with t(16;16)(p13.1;q22); CBFB-MYH11 | 46, XX, inv(16)(p13q22) | FLT3-TKD 9.75% mut； WT1+ | 2018/06/13 |
| 42 | F | 25 | | Acute monoblastic/monocytic leukemia | 48, XX, +X, +3, del(3))(p21)*2, -5, +8, del(8)(p11.2), del (8), t(8;11) (q22;q23); del(15)(q15q26.1); t(16;21)(p11.2;q22) | MLL-AF10+；KRAS 18.31% mut | 2018/06/19 |
| 43 | F | 62 | | Acute myelomonocytic leukemia | 46, XX | — | 2018/06/20 |
| 44 | F | 63 | | AML with mutated NPM1 | 46, XX | IDH2 44.53% mut；NPM1 16.32% mut | 2018/06/22 |
| 45 | M | 30 | | Acute monoblastic/monocytic leukemia | 46, XY | PHF6 67.41% mut; ASXL1 47.02% mut | 2018/06/26 |
| 46 | M | 49 | | AML with t(8;21)(q22;q22); RUNX1-RUNX1T1 | 45, X, -Y, t(6;21,8)(p25;q22;q22) | AML1-ETO+ | 2018/07/17 |
| 47 | M | 65 | | Acute myelomonocytic leukemia | 46, XY | RUNX1 42.82% mut; DNMT3A 40.87% mut; NRAS 4.97% mut; KRAS 6.6% mut; FLT3-ITD <50% mutation | 2018/07/26 |
| 48 | M | 26 | | Acute myelomonocytic leukemia | 46, XY | — | 2018/07/30 |
| 49 | F | 62 | | Acute monoblastic/monocytic leukemia | 46, XX | — | 2018/08/01 |
| 50 | F | 61 | | AML with maturation | 46, XX | TP53 37.73% mut; CEBPA 20.62% single-mut | 2018/08/01 |
| 51 | M | 42 | | AML with t(8;21)(q22;q22); RUNX1-RUNX1T1 | 48, XY, +4, +8, t(8;21)(q22;q22) | AML1-ETO+ | 2018/08/03 |
| 52 | M | 75 | | Acute monoblastic/monocytic leukemia | 45, X, -Y | — | 2018/08/20 |
| 53 | M | 37 | | AML with t(8;21)(q22;q22); RUNX1-RUNX1T1 | 46, XY | IDH2 40.67% mut；ASXL1 13.46% mut; NRAS 7.28%, 4.10% mut | 2018/08/23 |
| 54 | M | 50 | | AML with t(16;16)(p13.1;q22); CBFB-MYH11 | 46, XY, inv(16)(p13q22) | CBFB-MYH11+ | 2018/09/07 |
| 55 | M | 70 | | Acute monoblastic/monocytic leukemia | 86, XXYY, -2,-3,-5,-7,-16,-16 | — | 2018/09/07 |
| 56 | M | 45 | | Acute monoblastic/monocytic leukemia | 46, XY | IDH2 34.25% mut；WT1+ | 2018/10/09 |
| 57 | M | 20 | | AML with t(8;21)(q22;q22); RUNX1-RUNX1T1 | 45, X, -Y, t(8;21)(q22;q22) | WT1+；AML1-ETO+ | 2018/10/19 |
| 58 | M | 48 | | Acute monoblastic/monocytic leukemia | 48, XY, +8, +8 | TET2 50.18% mut | 2018/10/22 |
| 59 | M | 66 | | Acute monoblastic/monocytic leukemia | 46, XY | WT1+ | 2018/11/07 |
| 60 | F | 67 | | Acute myelomonocytic leukemia | 46, XX | SF3B1 39.69%, 1.27% mut；DNMT3A 41.2% mut; KRAS 11.09% mut | 2018/11/23 |
| 61 | F | 55 | | AML with maturation | 47, XX, +mar | WT1+；IDH1 24.43% mut | 2018/12/03 |
| 62 | M | 65 | | Acute myelomonocytic leukemia | 46, XY | FLT3-TKD+; FLT3-ITD= ins70bp <50% mut | 2018/12/07 |
| 63 | M | 45 | | AML with mutated NPM1 | 46, XY | NPM1 37.54% mut；DNMT3A 46.44% mut; FLT3-TKD+; NRAS 13.4% mut | 2018/12/07 |
| 64 | M | 40 | | Acute monoblastic/monocytic leukemia | 46, XY | DNMT3A 7.46% mut；PTPRT 46.04% mut; FBXW7 52.03% mut; TTN 47.62% mut | 2018/12/12 |
| 65 | M | 64 | | Acute myelomonocytic leukemia | 46, XY, t(7;11)(q11.2;p15) | Nup98-HoxA13+; Nup98-HoxC11+, Nup98-HoxD13+, Nup98-HoxA9+; Nup98-PMX1+; WT1+; Nup98-HoxA11+ | 2018/12/14 |

*：— means these patients have no molecular aberrations.

**Table S2. Multivariate analysis of patients with AML for survival.**

| Characteristics |  | OS |  | |  | |  | | DFS | |  | |  |
| --- | --- | --- | --- | --- | --- | --- | --- | --- | --- | --- | --- | --- | --- |
|  | OR | 95% CI | | *P* value | |  | | OR | | 95% CI | | *P* value | |
| PD-L1 Expression (≤7.39 vs. >7.39) | 0.853 | 0.354-2.054 | | 0.722 | |  | | 0.707 | | 0.253-1.976 | | 0.509 | |
| Frequency of Treg (≤10.35 vs. >10.35) | 0.570 | 0.240-1.355 | | 0.203 | |  | | 0.457 | | 0.164-1.270 | | 0.133 | |
| Frequency of PD-1^+^Treg (≤8.07 vs. >8.07) | 0.578 | 0.252-1.328 | | 0.197 | |  | | 0.483 | | 0.179-1.303 | | 0.151 | |
| Risk stratification (Favorable vs. Adverse) | 0.168 | 0.034-0.825 | | 0.028 | |  | | 0.214 | | 0.040-1.146 | | 0.072 | |
| Risk stratification (Intermediate vs. Adverse) | 0.396 | 0.057-0.925 | | 0.032 | |  | | 0.416 | | 0.151-1.141 | | 0.088 | |
| Risk stratification (Favorable vs. Intermediate) | 2.363 | 0.532-10.503 | | 0.259 | |  | | 1.946 | | 0.420-9.013 | | 0.395 | |
